# Supplementary material for: Health professionals’ competence for the provision of quality primary health care in Amhara region, Ethiopia
Source: PLoS One. 2025 Mar 4;20(3):e0315415. doi: 10.1371/journal.pone.0315415 (PMC11878939; doi:10.1371/journal.pone.0315415)
Supplement: Attachment — Submitted filename: Response letter.docx [file pone.0315415.s002.docx]

Dear Editor and Reviewers,

*Thank you so much for your kind words and valuable comments, as well as the opportunity to revise the manuscript. Your feedback has greatly improved our work. We have revised the manuscript according to your suggestions and recommendations.*

Reviewer #1: Author should explain more convincingly the sample technique. Apart from this, another limitation with the competency assessment technique. Competency assessment relying upon the self reported questionnaire can only satisy the Knowledge component of competency, in that case what about attitude and skills.

*Response: Thank you for your kind words and insightful comment. The sampling technique is described in lines 166-173. The competence of health professionals was measured using 30 competency statements that integrated knowledge, attitude, and skill components. This method aimed to assess the health professionals' perceptions of their performance on each competency described in the global competency framework for universal health coverage (WHO, 2022). Understanding their perceptions can inform policies to address competency gaps. They rated their competence and identified gaps in each statement, indicating a need for training. In the global competency framework for universal health coverage, "competent" refers to an individual’s ability to perform designated practice activities to a defined standard, which means possessing the requisite competencies. Competencies are defined as the abilities to integrate knowledge, skills, and attitudes in performing tasks in a given context (WHO,2022). Health professionals were asked whether they performed each competency statement by integrating these three components, not just knowledge. Moreover, this limitation is described in the discussion section of the manuscript, in lines 429-431.*

Reviewer #2: The article is based on the World Health Organisation's (WHO) Global Competency Framework for Universal Health Coverage (GCUHC), and the research is closely related to international topics, which to some extent reflects the current issues facing health system challenges. The article explores the primary health care capacity of health professionals in Amhara Region, Ethiopia. A mixed-methods research design was used, including both quantitative and qualitative data, and the methods of data collection and analysis, including logistic regression modelling and thematic analysis, as well as audio-recording and cross-checking were described in detail to increase the credibility of the findings. The article's findings show that only 14 per cent of health professionals consider themselves to have all competency areas, revealing important gaps in professional competence. The article's results point to the fact that primary health care competencies are strongly associated with factors such as educational status, training, taking licensure/competency certificate exams, and monthly salary, findings that are critical for developing strategies for improvement. The article also suggests that this study addresses contemporary emerging competency issues, that there was a lack of self-perception on the part of the respondents, and that the results may not be accurate.

*Response: Thank you for your kind words and insightful comments. The focus is to examine health professionals' perceptions through self-rating of each competency statement/item, allowing us to hear their voices on these competencies. This self-assessment is substantiated by a qualitative exploration of their competence.*

Recommendations:
Firstly, for the qualitative data sample of 12 technical professionals, the authors need to be mindful of whether more detailed information is provided on the qualitative data collection and analysis methods, including semi-structured questions for the interviews and specific steps for data analysis.

*Response: Thank you for your kind words and insightful comments. We have made revisions based on your comments, as indicated in lines 225-230 and 265-272 of the revised manuscript.*
Secondly, the study relied on health professionals' self-assessments to measure competence, which may be subject to social desirability bias, and participants may have overestimated or underestimated their competence. It is recommended to add a third-party perspective to the assessment results.

*Thank you for your kind words and insightful comments. To address the risk of social desirability bias, we used a self-administered questionnaire in line 222, allowing respondents to rate their competence at their convenience. Additionally, the quantitative data is supported by a qualitative exploration of their competence. By recogning this, we have included this as a limitation in the discussion section of the revised manuscript, as indicated in line 429-431. Furthermore, perspectives from service users, development partners, and health managers are included in another paper as part of a Ph.D. research project. Together, these papers will provide a holistic view of health professionals' competence*.

Third, the results of the article showed that only 14% of health professionals considered themselves to have all competency domains, and the results section should discuss the specific scores and distribution of each competency domain in more detail.

*Response: Thank you for your kind words and insightful comments. We have revised as per the comments as indicted in lines 313-317 in the revised version along with Table 2.*
Fourth, the discussion section suggests listing the consistency and discrepancy of the results with global and regional health policies.

*Response: Thank you for your kind words and insightful comments. We discussed the findings in relation to Universal Health Coverage and the philosophy of primary health care, which are local, regional, and global health policies, in lines 382-383 and 424-426 of the revised version.*
